# The value of ROX index in predicting the outcome of high flow nasal cannula: a systematic review and meta-analysis

**DOI:** 10.1186/s12931-022-01951-9

**Published:** 2022-02-17

**Authors:** Zhen Junhai, Yan Jing, Cao Beibei, Li Li

**Affiliations:** 1 Department of Critical Care Medicine, Zhejiang Hospital, Hangzhou, 310013 Zhejiang China; 2 Department of Critical Care Medicine, Zhejiang Hospital, Hangzhou, 310013 Zhejiang China; 3 Department of Pathology, Zhejiang Hospital, Hangzhou, 310013 Zhejiang China

**Keywords:** Acute respiratory failure, COVID-19, Pneumonia, High flow nasal cannula, ROX index

## Abstract

**Background:**

High flow nasal cannula (HFNC) therapy is widely employed in acute hypoxemic respiratory failure (AHRF) patients. However, the techniques for predicting HFNC outcome remain scarce.

**Methods:**

PubMed, EMBASE, and Cochrane Library were searched until April 20, 2021. We included the studies that evaluated the potential predictive value of ROX (respiratory rate-oxygenation) index for HFNC outcome. This meta-analysis determined sensitivity, specificity, positive likelihood ratio (PLR), negative likelihood ratio (NLR), diagnostic score, diagnostic odds ratio (DOR), and pooled area under the summary receiver operating characteristic (SROC) curve.

**Results:**

We assessed nine studies with 1933 patients, of which 745 patients experienced HFNC failure. This meta-analysis found that sensitivity, specificity, PLR, NLR, diagnostic score, and DOR of ROX index in predicting HFNC failure were 0.67 (95% CI 0.57–0.76), 0.72 (95% CI 0.65–0.78), 2.4 (95% CI 2.0–2.8), 0.46 (95% CI 0.37–0.58), 1.65(95% CI 1.37–1.93), and 5.0 (95% CI 4.0–7.0), respectively. In addition, SROC was 0.75 (95% CI 0.71–0.79). Besides, our subgroup analyses revealed that ROX index had higher sensitivity and specificity for predicting HFNC failure in COVID-19 patients, use the cut-off value > 5, and the acquisition time of other times after receiving HFNC had a greater sensitivity and specificity when compared to 6 h.

**Conclusions:**

This study demonstrated that ROX index could function as a novel potential marker to identify patients with a higher risk of HFNC failure. However, the prediction efficiency was moderate, and additional research is required to determine the optimal cut-off value and propel acquisition time of ROX index in the future.

PROSPERO registration number: CRD42021240607.

## Introduction

High flow nasal cannula oxygen therapy (HFNC) is designed to supply constantly inspired oxygen concentration (21–100%), temperature (31–37 °C), and humidity through high flow (8–80 L/min) nasal prongs. Although it is applied in clinics for only a short time, it has become a critical oxygen therapy tool for hypoxemic acute respiratory failure (ARF) patients nowadays. Several recently published studies have confirmed that HFNC is safe and effective for ARF patients [1–3], particularly for decreasing the intubation risk. Roca et al. [4] identified that HFNC was the only variable associated with a lower risk of subsequent mechanical ventilation (MV) (odds ratio 0.11, 95% CI 0.02–0.69; *P* = 0.02) in ARF patients. Another study demonstrated that patients with PaO_2_/FIO_2_ ≤ 200 mmHg exhibited a lower risk of intubation than standard oxygen therapy or non-invasive ventilation (NIV) [5].

As a previous study demonstrated that delaying intubation increases mortality [6], patients with a higher risk of HFNC failure must be carefully monitored, and intubation indications should be dynamically evaluated. However, identifying patients with a high risk of HFNC failure is a significant challenge. Higher respiratory rate and lower PaO_2_/FIO_2_ were two predictors of intubation in ARF patients treated with non-invasive oxygenation strategy [7], and since a great linear correlation is present between PaO_2_/FiO_2_ and SpO_2_/FiO_2_ parameters [8], SpO_2_/FiO_2_ can be an effective substitute for PaO_2_/FiO_2_. Based on the above principles, a new index called ROX (respiratory rate-oxygenation, calculated by the ratio of SpO_2_/FiO_2_ to respiratory rate) was developed in recent years. Several studies thought that ROX index had a good predictive capacity for HFNC outcome [9–14], whereas others demonstrated that ROX index should not be routinely utilized to predict HFNC outcome [15]. As a result, we conducted a systemic review and meta-analysis to systematically investigate the predictive capacity of ROX index for HFNC outcome.

## Methods

### Protocol and guidance

Our meta-analysis was conducted according to Preferred Reporting Items for Systematic Reviews and Meta-Analysis (PRISMA) [16]. The protocol of this study was registered, with PROSPERO registration number of CRD42021240607.

### Literature search strategy

According to the criteria of literature retrieval strategies, two authors independently searched MEDLINE, EMBASE, and Cochrane Library electronic databases. Since the first relevant article was published in 2016, the retrieval time was from 2016 to April 20, 2021. Without language restrictions, we conducted a search using the following terms: respiratory rate-oxygenation/ROX/ROX index/ROXI and HFNC/high-flow nasal cannula/nasal high-flow oxygen therapy/high-flow oxygen therapy. The study research disagreements were resolved by discussions, and when discussions failed to address the disagreements, a third author was consulted.

### Eligibility criteria

Literatures were selected based on the following eligibility criteria: (1) Studies evaluated the ability of ROX index in predicting HFNC outcome among adult patients (age over 18 years). (2) Number of true-positive (TP), true-negative (TN), false-positive (FP), and false-negative (FN) could be found or calculated by other data from the study. (3) No restrictions were set regarding the races or locations of patients and the types of diseases for which HFNC was employed. We excluded systematic reviews, case reports, pediatric studies, and repeated reports.

### Data extraction

Two data collectors independently read the literatures according to inclusion and exclusion criteria and extracted the associated data elements from the enrolled papers using a self-made data extraction form. Inconsistencies were solved by discussion, and when discussion failed to address the disagreements, a third author was consulted. The extracted contents were as follows: (1) The basic characteristics of the included studies: name of the first author, publication year, locations of the study, the diseases that require HFNC use, number of patients, the study design, acquisition time of ROX index and definition of HFNC failure in each enrolled study. (2) Other essential data of each study include TP, TN, FP, FN, cut-off value of ROX index, as well as the sensitivity and specificity to predict HFNC failure. We also contacted the corresponding authors by email when necessary data were not included in the article.

### Methodological quality assessment

Based on quality assessment of diagnostic accuracy studies 2 (QUADAS-2) guidelines [17], two authors independently assessed the risk of bias in enrolled studies, and a third author was invited to resolve disagreements during methodological quality assessment. The two main domains included risk of bias and applicability concerns. Each study evaluated the domain of risk of bias by evaluating patient selection, index test, reference standard, flow, and timing, whereas the domain of applicability concerns evaluated patient selection, index test, and reference standard. For each item, the risk of bias was classified as high, low, or unclear.

### Statistical analysis

The data analysis was conducted using STATA version 1.4 (Stata Corporation, College Station, TX). This meta-analysis determined pooled sensitivity, specificity, negative likelihood ratio (NLR), positive likelihood ratio (PLR), diagnostic score, and diagnostic odds ratio (DOR). When statistical heterogeneity was not found (*P* > 0.05, I^2^ < 50), the inconsistency factor (I^2^) statistics and Cochran-Q test were employed to analyze the potential heterogeneity, and the fixed-effect model was applied to calculate the pooled effect size; otherwise, the random-effects model was utilized. Subgroup analyses were employed to explain source of heterogeneity, and they included the types of diseases associated with HFNC use, cut-off value, and the acquisition time of ROX index. In addition, Deeks’ funnel plot was performed to estimate the potential publication bias.

## Results

### Literature search results

**Fig. 1:**
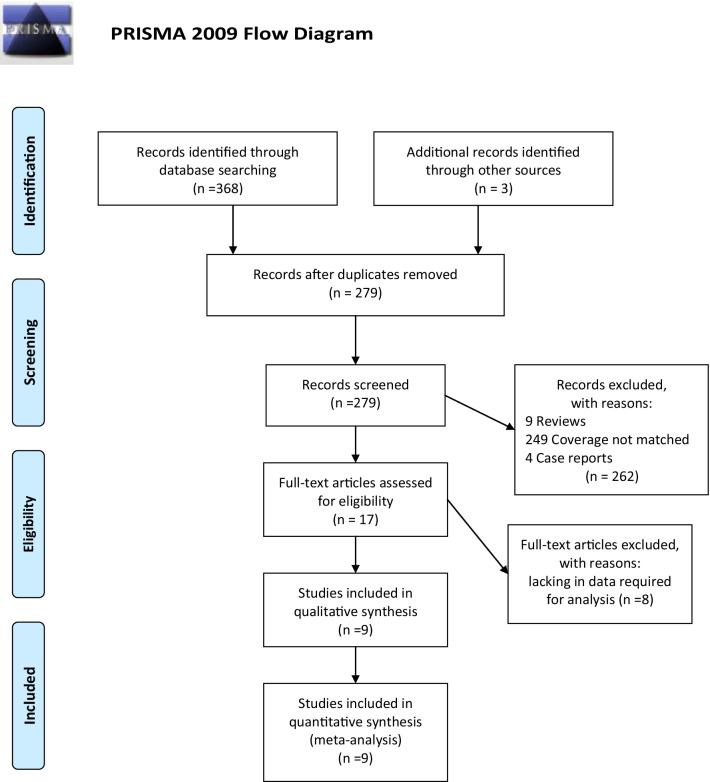
PRISMA flow diagram of the study selection process. From: Moher D, Liberati A, Tetzlaff J, Altman DG, The PRISMA Group (2009). Preferred reporting items for systematic reviews and meta-analyses: the PRISMA statement. PLoS Med 6(7): e1000097. 10.1371/journal.pmed1000097. For more information, visit www.prisma-statement.org

A total of 371 literatures were searched, including 260 from PubMed, 65 from EMBASE, and 46 from the Cochrane Library databases, 3 from other sources (such as abstracts from conferences). There were 279 articles left after excluding the reduplicated articles. After reviewing the abstracts, some articles were excluded, including systematic reviews, case reports, and coverage not matched, and 17 articles remained, which we read in full. A total of 8 studies were excluded due to the lack of data required for analysis, whereas 9 articles met the full inclusion criteria finally. The detailed PRISMA flowchart is depicted in Fig. 1.

### Characteristics of the enrolled trials

The detailed baseline characteristics are demonstrated in Tables 1 and 2. All the included trials were published between 2016 and 2021. Four studies were conducted in Europe [12–15], three in Asia [10, 11, 18], and the remaining two in USA [9] and Africa [19]. Four studies were multiple-center [12, 13, 15, 19] and the other five were single-center studies [9–11, 14, 18]. For the types of diseases requiring HFNC use, three studies were ARF [10, 14, 15], four were COVID-19 [9, 11, 18, 19], and two were pneumonia [12, 13]. The total number of patients was 1933, of which 745 were failure cases. Among these nine studies, five were prospective observational cohort studies [9, 10, 12, 13, 19], three were conducted retrospectively [11, 14, 18], and only one was RCT [15]. The acquisition time of ROX index after applying HFNC varied in different studies; six studies [9–12, 15, 19] reported 6 h as the acquisition time, one study [13] chose 12 h, another study chose 4 h [18], and the remaining study measured ROX index before each attempt to separate from HFNC [14]. HFNC failure was defined according to the included studies, five studies [9, 10, 12, 13, 15, 18] defined HFNC failure as the subsequent need for invasive MV, while Hu et al. [11] defined HFNC failure as the need for NIV or IMV and/or death, 1 study [19] defined HFNC failure as the need for MV or death, another study [14] defined HFNC failure as requiring HFNC resumption, NIV initiation, intubation, or death. The cut-off values of ROX index ranged from 2.7 to 9.2, and while three studies [12, 13, 15] reported 4.88 as the best cut-off value, other researchers found the values of 3.66 [9], 5.55 [11], 5.8 [10], 9.2 [14], 2.7 [19], 5.31 [18] in each study, and the sensitivity and specificity of ROX index to predict HFNC failure were from 50 to 84%.

**Table 1:** Characteristics of the included studies

Study	Country	Population	Toal cases	Failure cases	Study design	Acquisition time	Definition of HFNC failure
Virginie Lemiale 2021	France + Belgium	Immunocompromised patients with ARF	302	115	RCT	6 h after receiving HFNC	The subsequent need for invasive MV
Oriol Roca 2016	Spain + France	Pneumonia	157	44	Prospective observational cohort study	12 h after receiving HFNC	The subsequent need for invasive MV
Oriol Roca 2019	Spain + France	Pneumonia	191	68	Prospective observational cohort study	6 h after receiving HFNC	The subsequent need for invasive MV
Ken Junyang Goh 2020	Singapore	Acute hypoxemic respiratory	99	45	Prospective observational cohort study	6 h after receiving HFNC	The subsequent need for invasive MV
Abhimanyu Chandel 2020	USA	COVID-19	272	108	Prospective observational cohort study	6 h after receiving HFNC	The subsequent need for invasive MV
Ming Hu 2020	China	COVID-19	105	40	Retrospective cohort study	6 h after receiving HFNC	Need for NIV or IMV and/or death
Maeva Rodriguez 2019	France	ARF	190	22	Retrospective cohort study	Before each separation attempt	Requiring HFNC resumption, NIV initiation, intubation, or death
Gregory L Calligaro 2020	South Africa	COVID-19	293	156	Prospective observational cohort study	6 h after receiving HFNC	Need for MV or death
Jiqian Xu 2020	China	COVID-19	324	147	Retrospective cohort study	4 h after receiving HFNC	The subsequent need for invasive MV

*HFNC* high flow nasal cannula; *ARF* acute respiratory failure; *RCT* randomized controlled study; *MV* mechanical ventilation; *NV* noninvasive ventilation, *IMV* invasive ventilation

**Table 2:** The detailed characteristics of the included studies

Study	TP	FP	TN	FN	Cut-off	Sen	Spe
Virginie Lemiale 2021	60	58	129	55	4.88	0.52	0.69
Oriol Roca 2016	31	31	82	13	4.88	0.70	0.73
Oriol Roca 2019	57	61	62	11	4.88	0.84	0.50
Ken Junyang Goh 2020	26	14	40	19	5.8	0.58	0.74
Abhimanyu Chandel 2020	53	26	138	55	3.66	0.49	0.84
Ming Hu 2020	34	25	40	6	5.55	0.85	0.62
Maeva Rodriguez 2019	11	27	141	11	9.2	0.50	0.84
Gregory L Calligaro 2020	106	32	105	50	2.7	0.68	0.77
Jiqian Xu 2020	114	60	117	33	5.31	0.78	0.66

*TP* true positive; *FP* false positive; *TN* true negative; *FN* false negative; *Sen* sensitivity; *Spe* specificity

### Results of methodological quality evaluation

After careful evaluation of the methodological quality of all nine enrolled studies, we found that seven studies [9, 10, 12, 13, 15, 18, 19] had a low risk of bias in patient selection, three trials [12, 13, 15] had a low risk of bias in index test, all the included studies exhibited low risk of bias in the item of reference standard, and eight studies [9–13, 15, 18, 19] had low risk in flow and timing. To sum up, the included seven studies were of good quality (Table 3).

**Table 3:** Methodological quality assessment of studies included

Study	Risk of bias				Applicability concerns		
	Patient selection	Index test	Reference standard	Flow and timing	Patient selection	Index test	Reference standard
Virginie Lemiale 2021	LR	LR	LR	LR	LC	LC	LC
Oriol Roca 2016	LR	LR	LR	LR	LC	UC	LC
Oriol Roca 2019	LR	LR	LR	LR	LC	LC	LC
Ken Junyang Goh 2020	LR	UR	LR	LR	LC	LC	LC
Abhimanyu Chandel 2020	LR	UR	LR	LR	LC	LC	LC
Ming Hu 2020	UR	UR	LR	LR	LC	LC	LC
Maeva Rodriguez 2019	UR	UR	LR	UR	LC	UC	LC
Gregory L Calligaro 2020	LR	UR	LR	LR	LC	LC	LC
Jiqian Xu 2020	LR	UR	LR	LR	LC	LC	LC

*LR* low risk; *HR* high risk; *UR* unclear risk; *LC* low concern; *HC* high concern; *UC* unclear concern

### The meta-analysis of the ability of ROX index to predict HFNC failure

**Fig. 2:**
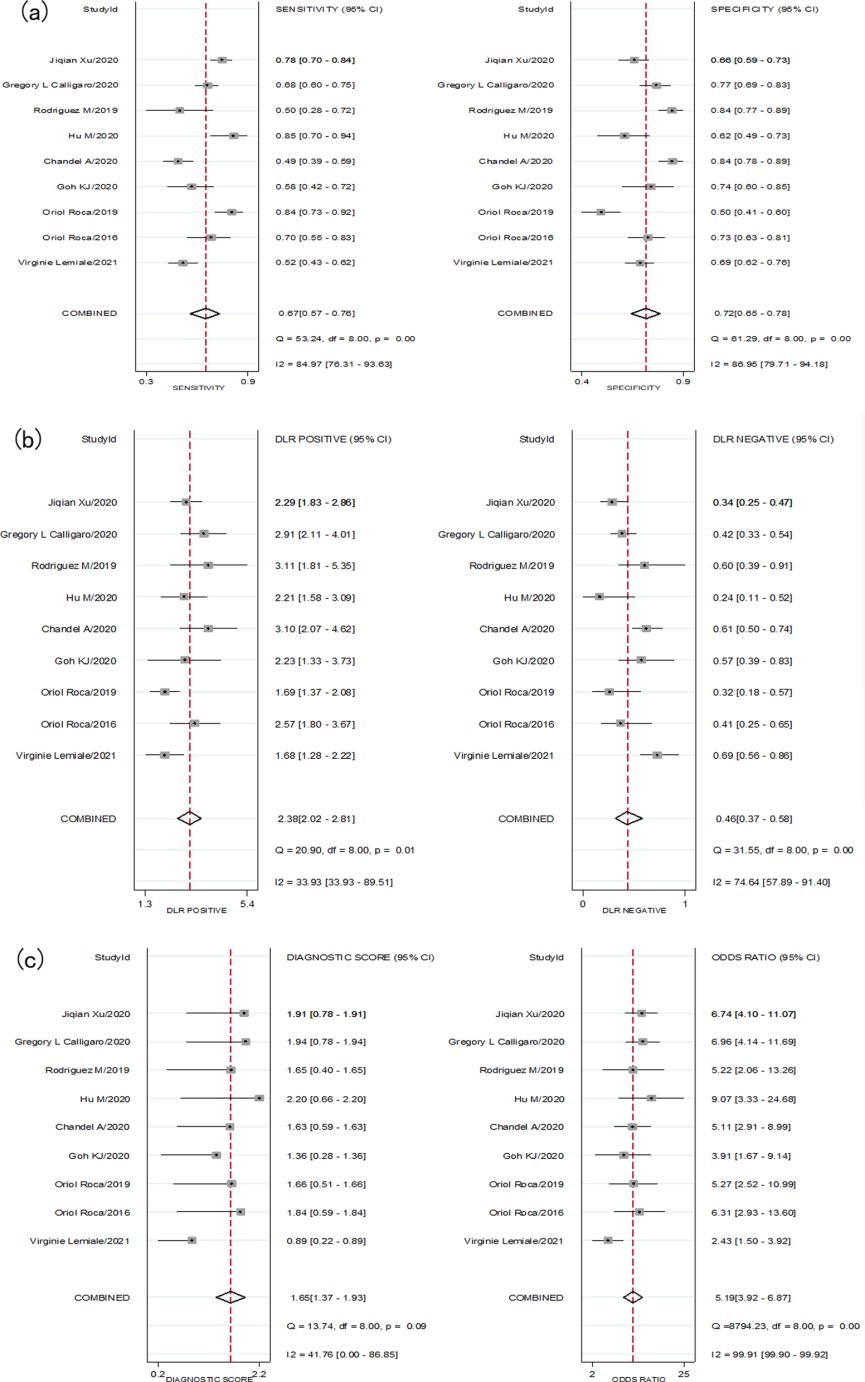
**a** Forest plot of the sensitivity (SEN) and specificity (SPE) of the ROX index in predicting outcome of High flow nasal cannula (HFNC), the pooled SEN and SPE were 0.67 (95% CI 0.57–0.76) and 0.72 (95% CI 0.65–0.78), respectively. **b** Forest plot of the positive likelihood ratio (PLR), negative likelihood ratio (NLR) of the ROX index in predicting outcome of HFNC, the pooled PLR and NLR were 2.4 (95% CI 2.0–2.8) and 0.46 (95% CI 0.37–0.58), respectively. **c** Forest plot of the diagnostic score, diagnostic odds ratio (DOR) of the ROX index in predicting outcome of HFNC, the pooled diagnostic score and DOR were 1.65(95% CI 1.37–1.93) and 5.0 (95% CI 4.0–7.0), respectively

**Fig. 3:**
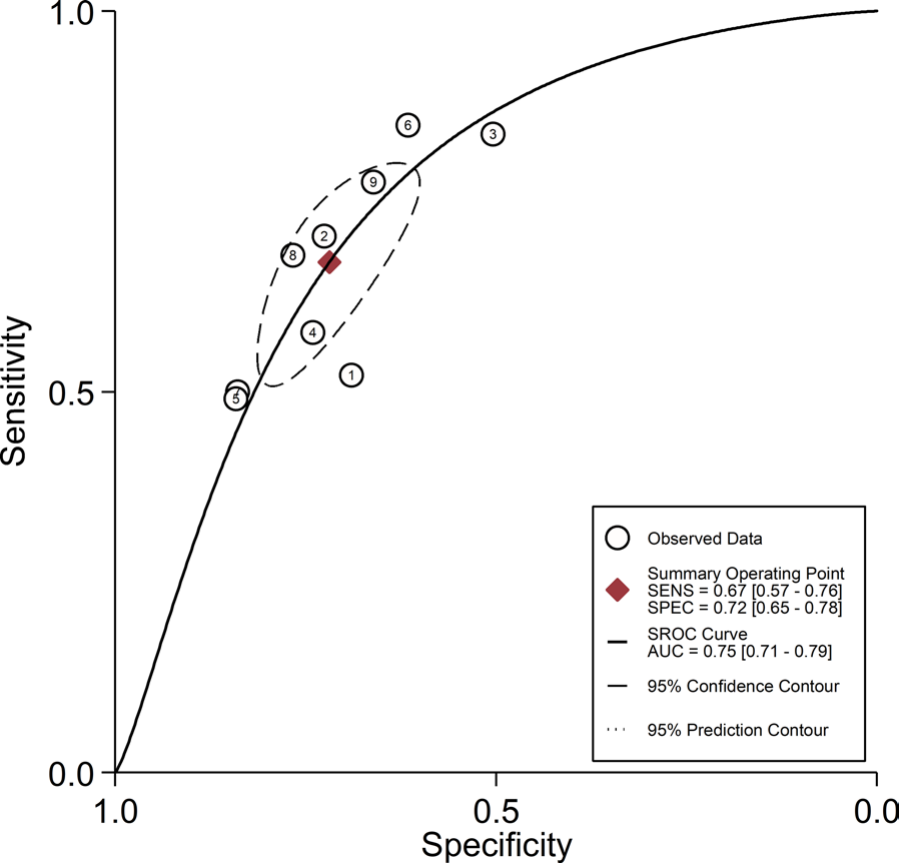
The summary receiver operating characteristic (SROC) plot to evaluate the predicting accuracy of ROX index, and the area under the curve (AUC) was 0.75 (95% CI 0.71–0.79), implying that ROX index could predict HFNC failure

Nine trials with 1933 patients assessed the ability of ROX index in predicting HFNC outcome, and there was statistically significant heterogeneity in the sensitivity (*I*^2^ = 84.97%), specificity (*I*^2^ = 86.95%), PLR (*I*^2^ = 33.93%), NLR (*I*^2^ = 74.64%), diagnostic score (*I*^2^ = 41.76%) and DOR (*I*^2^ = 99.91%). Therefore, we used a random-effect model to conduct this meta-analysis. Pooling all the enrolled studies, the sensitivity, specificity, PLR, NLR, diagnostic score, and DOR of ROX index in predicting the HFNC failure are depicted in Fig. 2. The pooled estimates of ROX index in predicting HFNC failure were as follows: sensitivity, 0.67 (95% CI 0.57–0.76); specificity, 0.72 (95% CI 0.65–0.78); PLR, 2.4 (95% CI 2.0–2.8); NLR, 0.46 (95% CI 0.37–0.58); diagnostic score, 1.65(95% CI 1.37–1.93); and DOR, 5.0 (95% CI 4.0–7.0). We also conducted the summary receiver operating characteristic (SROC) plot to evaluate the predicting accuracy of ROX index (Fig. 3), and the area under the curve (AUC) was 0.75 (95% CI 0.71–0.79), implying that ROX index could predict HFNC failure.

### Subgroup analyses for the predicting value of ROX stratified by different conditions

To determine heterogeneity between studies, we conducted subgroup analyses based on the types of diseases correlated with HFNC use, the areas of enrolled studies, and the acquisition time of ROX index (Table 4). For the types of diseases correlated with HFNC use, there were four trials reported data on COVID-19, with pooled sensitivity and specificity of 0.71 (0.56–0.82) and 0.73 (0.63–0.81), respectively, while lower sensitivity (0.63, 0.48–0.75) and specificity (0.71, 0.60–0.80) were found among other diseases. In the subgroup of cut-off value, the sensitivity and specificity in predicting HFNC failure were 0.59 (0.54–0.65), 0.83 (0.79–0.86) when the cut-off value > 5, and 0.67 (0.65–0.76), 0.71 (0.61–0.80) in ≤ 5 cut-off value. The pooled results of six enrolled studies indicated that summary sensitivity and specificity were 0.67 (0.54–0.78) and 0.70 (0.60–0.78) when acquisition time was 6 h after receiving HFNC. However, good performance of ROX index was found in settings with other acquisition times, with sensitivity and specificity of 0.73 (0.67–0.79) and 0.74 (0.70–0.78), respectively.

**Table 4:** Subgroup analysis for the predicting value of ROX

Subgroups	Number of articles	Sen	Spe	PLR	NLR	DOR	AUC
Overall studies	9	0.67 (0.57–0.76)	0.72 (0.65–0.78)	2.4 (2.0–2.80)	0.46 (0.37–0.58)	5 (4–7)	0.75 (0.71–0.79)
Population							
COVID-19	4	0.71 (0.56–0.82)	0.73 (0.63–0.81)	2.60 (2.10–3.30)	0.40 (0.28–0.56)	7 (5–9)	0.78 (0.74–0.82)
Other population	5	0.63 (0.48–0.75)	0.71 (0.60–0.80)	2.10 (1.70–2.70)	0.53 (0.40–0.70)	4 (3–6)	0.72 (0.68–0.76)
Acquisition time							
6 h after receiving HFNC	6	0.67 (0.54–0.78)	0.70 (0.60–0.78)	2.20 (1.80–2.80)	0.47 (0.35–0.63)	5 (3–7)	0.74 (0.70–0.78)
Other time	3	0.73 (0.67–0.79)	0.74 (0.70–0.78)	2.84 (2.39–3.39)	0.36 (0.29–0.45)	7 (5–10)	0.79 (0.75–0.83)
Cut-off value							
Cut-off ≤ 5	5	0.67 (0.65–0.76)	0.71 (0.61–0.80)	2.3 (1.8–2.9)	0.48 (0.37–0.63)	5 (3–7)	0.74 (0.70–0.78)
Cut-off > 5	4	0.59 (0.54–0.65)	0.83 (0.79–0.86)	3.5 (2.78–4.43)	0.49 (0.43–0.56)	7 (5–9)	0.78 (0.74–0.82)

*Sen* sensitivity; *Spe* specificity; *PLR* positive likelihood ratio; *NLR* negative likelihood ratio; *DOR* diagnostic odds ratio; *AUC* area under curve; *HFNC* high flow nasal cannula

### Publication bias analysis

**Fig. 4:**
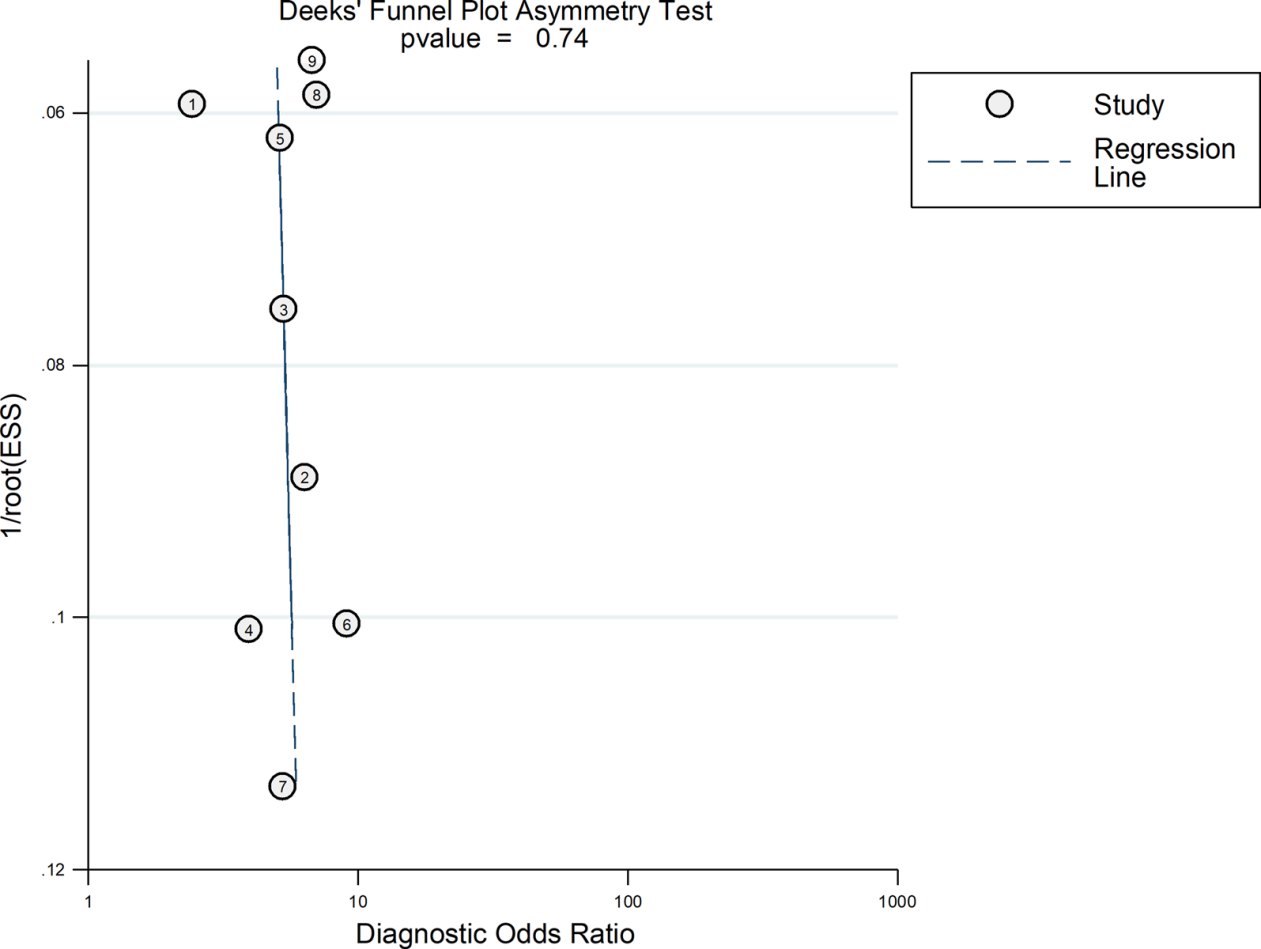
The Deeks’ funnel plot to evaluate the potential publication bias, this funnel plot demonstrated no statistically significant publication bias in this meta‐analysis (*P* = 0.74)

We performed Deeks’ funnel plot to evaluate the potential publication bias, and the funnel plot demonstrated no statistically significant publication bias in this meta‐analysis (*P* = 0.74) (Fig. 4).

## Discussion

The accurate prediction of HFNC outcome ensures timely detection of patients with a higher risk of intubation. This study evaluated ROX index for sensitivity and specificity in predicting HFNC failure. Our meta-analysis revealed that pooled sensitivity and specificity of ROX index were 0.67 (95% CI 0.57–0.76) and 0.72 (95% CI 0.65–0.78), respectively, the overall value of ROX index had an acceptable specificity, whereas the sensitivity was low. In the subgroup analysis, our study demonstrated that ROX index was more predictive in COVID-19 patients, cut-off value > 5, and other acquisition times (compared with 6 h after receiving HFNC). Consequently, we believed that ROX index is a promising marker to identify patients with a higher risk of HFNC failure, with a moderate prediction efficiency.

This meta-analysis demonstrated that ROX index could be a viable tool for clinicians to assess HFNC progress and outcome, as confirmed by many previous studies. However, because there is certain degree of heterogeneity in patients with respiratory failure caused by different types of diseases, in subgroup analysis, we conducted subgroup analysis on patients in different populations, divided them into COVID-19 group and other population group (pneumonia and ARF), and the result showed higher discriminatory accuracy among COVID-19 group compared to other population group (pneumonia and ARF). However, the number of studies is too small for non-COVID-19 patients with only two studies about pneumonia patients and three studies about ARF patients. Furthermore, most ARF patients were caused by pneumonia (but the specific data are not explained in detail in some studies, we also tried to contact the researchers to ask for specific data, but in vain), Therefore, we did not conduct further subgroup analysis on other population (pneumonia and ARF), which may disturb the result of our meta-analysis. We thought more studies are needed to further analyze the predictive value of ROX among non-COVID-19 patients.

There is no universal agreement on the cut-off value of the ROX index, so the cut-off values used in the included studies varied from 2.7 to 9.2, we further conducted the subgroup meta-analysis stratified by cut-off value, and our subgroup analysis demonstrated higher discriminatory accuracy including studies used cut-off value > 5 compared to ≤ 5 cut-off value, thus, we thought > 5 cut-off value maybe close to the optimal cut-off value. For the acquisition time of the ROX index, most studies [9–12, 15, 19] reported 6 h as the acquisition time, however, our subgroup meta-analysis demonstrated good performance of ROX index was found in settings with other acquisition times, for the number of studies enrolled was small about the other acquisition times of the ROX index, we thought the ideal time to acquire the index remains unknown, and further studies are required to find the proper time.

This meta-analysis illustrated that prediction efficiency of ROX index was moderate, and several reasons may explain this. On the one hand, the cut-off and the acquisition time of ROX index varied in different studies, and differences in study patients may cause variation in the ideal cut-off value and the acquisition time. On the other hand, the ROX index could only reflect the work of breathing, and other factors such as tachycardia were associated with HFNC failure [7]. The prediction ability of ROX index may be improved when combined with other parameters. In 2020, Goh et al. [10] reported a new index called ROX-HR index (the ratio of ROX index over HR), and their study indicated that ROX-HR index higher than eight was significantly associated with HFNC success at 6 and 10 h, and ROX-HR index outperformed ROX index in predicting HFNC failure among postintubation patients. However, this was the only study that evaluated ROX-HR index value in predicting HFNC failure. Finally, the challenging problem is when to conduct intubation when ROX index is lower than the cut-off value. Should we act immediately or wait until the criteria to intubate are met completely? Obviously, studies are required to identify an optimal intubation time in the future.

However, this meta-analysis also has several limitations. Firstly, most studies included had a small sample size; this may distort the results to some extent. Secondly, only one study was multicenter RCT, whereas the most were single-center prospective observational cohort studies. Thirdly, there was significant heterogeneity among the enrolled studies. Although we conducted subgroup analysis to identify the source of heterogeneity, other factors like bias in HFNC failure definition, the lack of common intubation criteria between studies may also be possible heterogeneity sources, which may cause practice variation. As a consequence, since findings and interpretations of this meta-analysis are limited by shortcomings showed above, it is crucial to be prudent when referencing some results of this study and must consider personal experiences and practical situations of patients.

Our meta-analysis has the following strengths. Firstly, this was the first meta-analysis to systematically assess ROX index value for predicting HFNC failure among different patients. Secondly, we conducted several meaningful subgroup analyses to comprehensively evaluate ROX index value in predicting HFNC failure, this may help guide clinical practice. Thirdly, our study strictly conformed to the broad EQUATOR guidelines-a tool to ensure the value and reliability of research literature [20].

## Conclusion

In summary, our meta-analysis reveals that ROX index is an easy-to-use and promising tool for clinicians to identify patients with a higher risk of HFNC failure, and those with a lower value of ROX index must be carefully monitored, accompanied by dynamic evaluation of intubation indications. Additional studies are required to determine the best cut-off value and the proper acquisition time of ROX index in the future.

## Data Availability

Data sharing is not applicable to this article, as this study is a meta-analysis.

## References

[1] Roca O, Riera J, Torres F (2010). High-flow oxygen therapy in acute respiratory failure. Respir Care.

[2] Moretti M, Cilione C, Tampieri A (2000). Incidence and causes of non-invasive mechanical ventilation failure after initial success. Thorax.

[3] Bonnet N, Martin O, Boubaya M (2021). High flow nasal oxygen therapy to avoid invasive mechanical ventilation in SARS-CoV-2 pneumonia: a retrospective study. Ann Intensive Care.

[4] Roca O, De Acilu MG, Caralt B (2015). Humidified high flow nasal cannula supportive therapy improves outcomes in lung transplant recipients readmitted to the intensive care unit because of acute respiratory failure. Transplantation.

[5] Frat JP, Thille AW, Mercat A (2015). High-flow oxygen through nasal cannula in acute hypoxemic respiratory failure. N Engl J Med.

[6] Antonelli M, Conti G, Moro ML (2001). Predictors of failure of noninvasive positive pressure ventilation in patients with acute hypoxemic respiratory failure: a multi-center study. Intensive Care Med.

[7] Frat JP, Ragot S, Coudroy R (2018). Predictors of intubation in patients with acute hypoxemic respiratory failure treated with a noninvasive oxygenation strategy. Crit Care Med.

[8] Desprez K, McNeil JB, Wang C (2017). Oxygenation saturation index predicts clinical outcomes in ARDS. Chest.

[9] Chandel A, Patolia S, Brown AW (2020). High-flow nasal cannula therapy in COVID-19: using the ROX index to predict success. Respir Care.

[10] Goh KJ, Chai HZ, Ong TH (2020). Early prediction of high flow nasal cannula therapy outcomes using a modified ROX index incorporating heart rate. J Intensive Care.

[11] Hu M, Zhou Q, Zheng R (2020). Application of high-flow nasal cannula in hypoxemic patients with COVID-19: a retrospective cohort study. BMC Pulm Med.

[12] Roca O, Caralt B, Messika J (2019). An index combining respiratory rate and oxygenation to predict outcome of nasal high-flow therapy. Am J Respir Crit Care Med.

[13] Roca O, Messika J, Caralt B (2016). Predicting success of high-flow nasal cannula in pneumonia patients with hypoxemic respiratory failure: the utility of the ROX index. J Crit Care.

[14] Rodriguez M, Thille AW, Boissier F (2019). Predictors of successful separation from high-flow nasal oxygen therapy in patients with acute respiratory failure: a retrospective monocenter study. Ann Intensive Care.

[15] Lemiale V, Dumas G, Demoule A (2021). Performance of the ROX index to predict intubation in immunocompromised patients receiving high-flow nasal cannula for acute respiratory failure. Ann Intensive Care.

[16] Liberati A, Altman DG, Tetzlaff J (2009). The PRISMA statement for reporting systematic reviews and meta-analyses of studies that evaluate health care interventions: explanation and elaboration. Ann Intern Med.

[17] Whiting PF, Rutjes AW, Westwood ME (2011). QUADAS-2: a revised tool for the quality assessment of diagnostic accuracy studies. Ann Intern Med.

[18] Xu J, Yang X, Huang C (2020). A novel risk-stratification models of the high-flow nasal cannula therapy in COVID-19 patients with hypoxemic respiratory failure. Front Med.

[19] Calligaro GL, Lalla U, Audley G (2020). The utility of high-flow nasal oxygen for severe COVID-19 pneumonia in a resource-constrained setting: a multi-centre prospective observational study. EClinicalMedicine.

[20] Simera I, Moher D, Hoey J (2010). A catalogue of reporting guidelines for health research. Eur J Clin Invest.

